# Comparison of Magnetic Resonance Imaging and Computed Tomography for Evaluation of Cervical Vertebral Nerves and Synovial Tissues in Horses with Cervical Spinal Disease

**DOI:** 10.3390/ani16121759

**Published:** 2026-06-06

**Authors:** Alyssa M. Daniels, Alison J. Morton, Natasha M. Werpy, Adam H. Biedrzycki, Travis M. Tull, Thomas N. Denagamage, Erin G. Porter, Jennifer S. Taintor, Robin J. W. Bell

**Affiliations:** 1 Department of Large Animal Clinical Sciences, University of Florida College of Veterinary Medicine, Gainesville, FL 32610, USA; alyssa.daniels1@ufl.edu (A.M.D.); dradam@ufl.edu (A.H.B.); tdenagamage@ufl.edu (T.N.D.); gordone@ufl.edu (E.G.P.); jtaintor@ufl.edu (J.S.T.); robin.bell@ufl.edu (R.J.W.B.); 2 Equine Diagnostic Imaging, Inc., Archer, FL 32618, USA; natasha@eqdxim.com; 3 Ocala Equine Hospital, Ocala, FL 34482, USA; travistull@gmail.com

**Keywords:** equine, cervical, computed tomography, magnetic resonance imaging

## Abstract

Pathology of the cervical spine is an important cause of poor performance in sport horses. Computed tomography (CT) and magnetic resonance imaging (MRI) have improved musculoskeletal diagnostic capabilities. CT is well-suited to assess osseous pathology, although it is limited for assessing soft tissues. MRI, the gold standard for imaging soft tissues, cannot be used for antemortem imaging of the equine cervical spine. The diagnostic potential of CT for evaluating soft tissues of the equine cervical spine compared to MRI is not well-defined and warrants investigation. Postmortem MRI was performed on eighteen horses euthanized for clinical and CT myelographic findings of cervical disease. Synovial and vertebral nerve tissue abnormalities were graded on MRI and CT. Grading of radiologists’ confidence in identification of synovial tissues and vertebral nerves on MRI and CT was performed. Hounsfield unit (HU) measurements of synovial and vertebral nerve tissues were recorded. MRI was superior at evaluating, and radiologists had greater confidence in MRI for identifying synovial and vertebral nerve tissues. Agreement between measurements of HU was variable and poorest for vertebral nerves. Findings support MRI’s superiority for the evaluation of equine cervical vertebral nerves and synovial tissues. Caution should be exercised when interpreting CT findings of these tissues of horses.

## Simple Summary

Disorders of the cervical spine are a common cause of poor performance in sport horses, and these conditions can be challenging to diagnose. Magnetic resonance imaging (MRI) is used commonly and is the gold standard for imaging cervical soft tissues in people and other species; however, currently the necks of adult horses cannot be imaged because of the configuration and size of MRI units. Computed tomography (CT) is available and provides the most complete imaging of the cervical spine of horses, although it does not provide the same detail of important soft tissues as MRI. It is not well understood how MRI and CT compare for evaluating soft tissues in horses’ cervical spines, or how well CT can predict abnormalities in these tissues. This study compared cervical soft tissues on CT and MRI images from cadavers of horses that had been euthanized for cervical spinal disease. The results showed that MRI provided superior imaging to CT and that radiologists lacked confidence in their assessments of these tissues when using CT. These findings support the use of caution when interpreting cervical soft tissues of horses on CT and encourage development of MRI units that may accommodate the imaging of horses’ necks.

## 1. Introduction

Pathology of the equine cervical spine is increasingly recognized as an important contributor to poor performance and neurological deficits, particularly in sport horses [1]. Lesions involving the articular process joints, spinal cord, vertebral nerves, and perineural tissues may result in a range of clinical signs, often manifesting as cervical pain and stiffness, intermittent forelimb lameness, resistance to work or generalized performance decline [2,3,4]. These signs can be vague and nonspecific, frequently leading to diagnostic uncertainty during clinical and radiographic evaluations [5,6,7].

Conventional imaging modalities such as radiography and ultrasonography are more widely available in equine practice but provide an incomplete assessment of the cervical spine. Radiographs are limited by their two-dimensional representation of three-dimensional structures, leading to inadequate sensitivity and specificity for diagnosing cervical spinal pathology [8,9]. Ultrasonography provides a limited evaluation of articular process joints and periarticular soft tissues [6,10]. Advanced imaging modalities such as computed tomography (CT) and magnetic resonance imaging (MRI) have significantly enhanced diagnostic capabilities in equine medicine. Computed tomography provides excellent spatial resolution and is well-suited to the assessment of osseous pathology, including vertebral canal narrowing and osseous changes in the articular process joints [6,11,12]. Additionally, CT allows for objective quantification of tissue density using Hounsfield units (HU), which may aid in distinguishing different tissue types in both clinical and research settings [13,14]. Magnetic resonance imaging, by contrast, offers superior soft tissue contrast and is widely considered the gold standard for imaging soft tissue structures in both human and veterinary medicine [15,16]. It enables detailed assessment of spinal cord parenchyma, vertebral nerve and nerve roots, intervertebral discs and perineural tissues that are often not adequately identified on CT [15]. However, MRI cannot currently be used for antemortem imaging of the entire equine cervical spine due to the physical constraints of magnet bore size and coil designs.

Despite growing recognition of cervical spinal disease as a cause of neurological dysfunction and decreased performance, accurate identification of soft tissue abnormalities remains limited by the capabilities of currently available antemortem imaging modalities. Cervical vertebral malformations, dynamic compression, and degenerative changes have been documented with notable frequency in horses presenting with mild neurological signs or unexplained poor performance [3,4,17,18]. Nonetheless, the diagnostic potential of CT for evaluating soft tissue structures of the equine cervical spine compared to MRI is not well-defined and warrants further investigation.

The aim of this study was to compare the diagnostic utility of CT and MRI for the identification of soft tissue structures of the equine cervical spine, specifically the synovial tissues of the articular process joints, vertebral nerves and perineural tissues. The objectives were: (1) to describe and compare the distribution and severity of soft tissue abnormalities on CT and MRI in horses with cervical disease; (2) to evaluate the agreement of radiologists’ Hounsfield unit (HU) measurements of cervical synovial tissues and vertebral nerves on CT; and (3) to compare cervical spinal soft tissue pathology and radiologists’ confidence in their ability to identify cervical synovial tissues and vertebral nerves on MRI and CT. The hypotheses were as follows: (1) MRI will be superior to CT for identifying soft tissue pathology; (2) there will be moderate agreement between radiologists on HU measurements of cervical synovial tissues and vertebral nerves; and (3) radiologists will have greater confidence in MRI for the identification of cervical synovial tissues and vertebral nerves.

## 2. Materials and Methods

### 2.1. Inclusion Criteria

Cadaveric specimens from eighteen horses euthanized for clinical and CT myelographic findings of performance-limiting cervical spinal disease between February 2022 and March 2025 were included. Age, sex, and breed for each were recorded. Prior to CT myelographic examination, horses had a complete neurological examination completed by a veterinary specialist. Horses with a history of cervical trauma were excluded.

For the CT examination, horses were placed under general anesthesia in routine fashion and positioned in right lateral recumbency in the CT unit. Atlanto-occipital centesis was performed, and 50 mL of cerebrospinal fluid (CSF) was collected and replaced with 50 mL of 300 mg/mL Iohexol (Omnipaque Iohexol; GE Healthcare, Boston, MA, USA) contrast. Computed tomographic imaging was performed using an intermittent sequential mode with a 320-detector row CT scanner with a 160 mm wide-area (2D) solid-state detector design that allows image acquisition of a volumetric axial length of 160 mm without moving the CT (Aquilion; Canon Medical Systems, Tustin, CA, USA) couch. The gantry opening was 78 cm. A field-of-view of 50 cm with 512 × 512 pixels, 0.5 mm slice thickness and a tube rotation time of 0.35 s at 100 kVp and 280 mAs (100 effective mAs) was used [11]. Imaging was performed from the first cervical vertebra to the second thoracic vertebra in neutral, flexed, and extended positions.

### 2.2. Collections and Preparation of Specimens

Following euthanasia, the cadaveric head and neck was collected. The thoracic spine was disarticulated between the third and fourth thoracic vertebra, and the vertebral canal was simultaneously plugged with modeling compound (Play-Doh; Hasbro, Pawtucket, RI, USA) to limit leakage of CSF and entry of air that might interfere with MRI.

### 2.3. Magnetic Resonance Imaging

The specimens were placed in a 1.5 Tesla high-field MRI unit (1.5T MRI system; Canon Vantage Titan, Tustin, CA, USA; 1.5T MRI system; GE Signa EXCITE, Boston, MA, USA; 1.5T MRI system; Canon Vantage Orian, Tustin, CA, USA) in right lateral position with the head and neck in neutral position. The regions of abnormalities determined on CT myelography were imaged. Additional cervical regions were imaged if time permitted. T1-weighted (T1W), T2-weighted (T2W), T1-wieghted with fat saturation (T1-FS), and proton density (PD) sequences were used, and images were acquired using a phase array body coil in sagittal, axial, and dorsal planes (Table 1).

### 2.4. Image Assessment

All images were transferred, stored, and analyzed in Digital Imaging and Communications in Medicine (DICOM) format on Asteris Keystone Omni (Asteris Keystone Omni; Asteris Inc., Monument, CO, USA). Images were analyzed independently by two board-certified veterinary radiologists. Images with differing scores were reviewed again and scores discussed. A final score was then assigned by consensus between the two observers. All images were evaluated to assess each component, with certain sequences and planes more suitable for grading individual components than others.

### 2.5. Equine Cervical MRI Image Assessments

**Figure 1:**
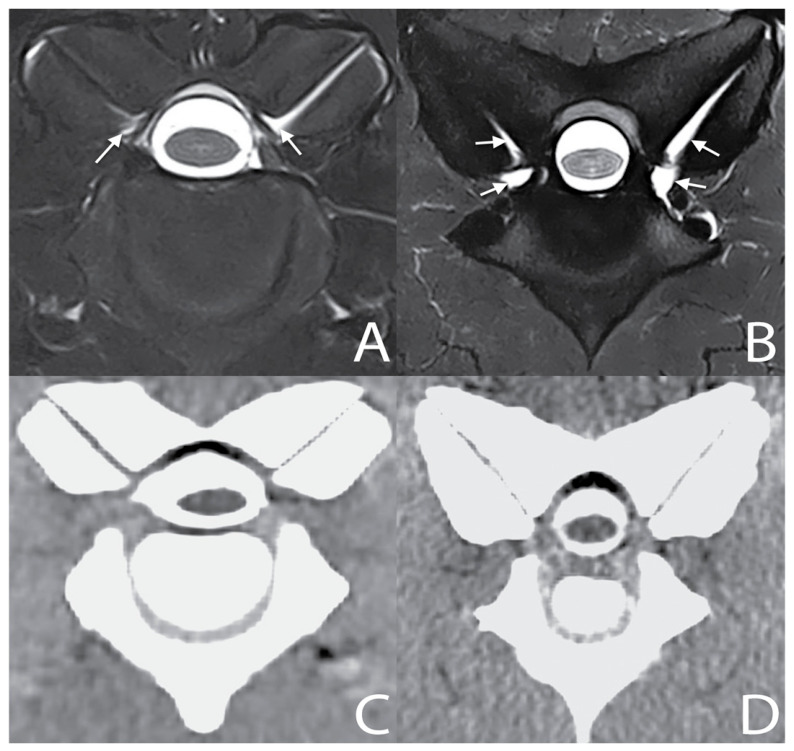
Comparison of MRI synovial effusion grades and corresponding CT images. (**A**) T2W MRI of C3-C4 with grade 1 (left arrow) and grade 2 (right arrow) synovial effusion. (**B**) T2W MRI of C4-C5 of grade 3 (left arrows) and grade 4 (right arrows) synovial effusion. (**C**) CT image of same horse and sites as in MRI image (**A**). (**D**) CT image of same horse and sites as in MRI image (**B**).

**Figure 2:**
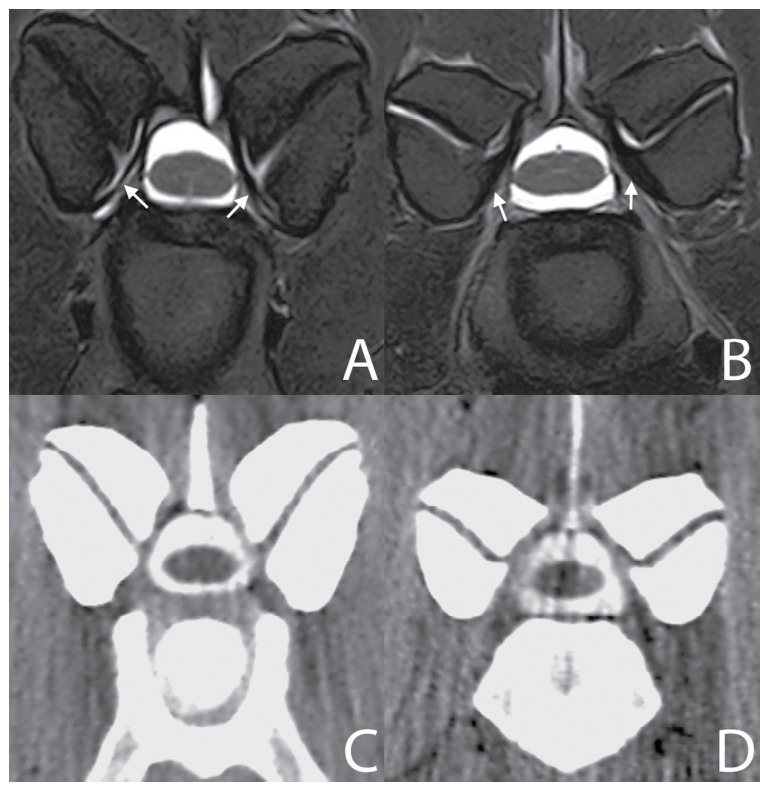
Comparison of MRI synovial membrane thickening and corresponding CT images. (**A**) T2W MRI of C6-C7 with grade 1 (left arrow) and grade 2 (right arrow) synovial membrane thickening. (**B**) T2W MRI of C7-T1 of grade 3 (left arrow) and grade 4 (right arrow) synovial membrane thickening. (**C**) CT image of same horse and sites as in MRI image (**A**). (**D**) CT image of same horse and sites as in MRI image (**B**).

**Figure 3:**
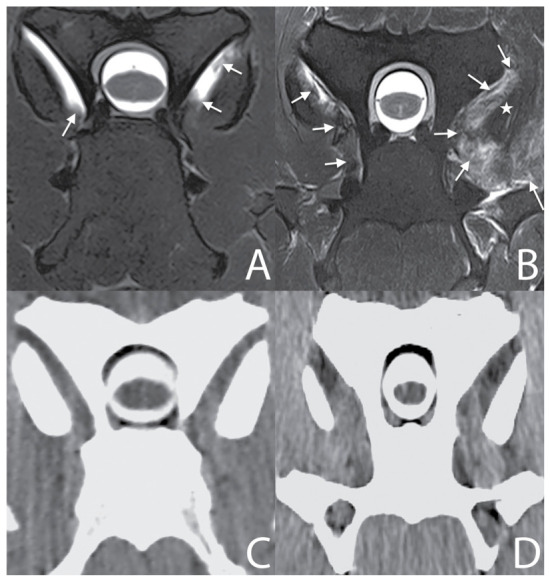
Comparison of MRI synovial proliferation and corresponding CT images. (**A**) T2W MRI of C6-C7 with grade 1 (left arrow) and grade 2 (right arrows) synovial proliferation. (**B**) T2W MRI of C6-C7 of grade 3 (left arrows) and grade 4 (right arrows) synovial proliferation. Cranial aspect of C7 (star) defining C6-C7 joint space with extension of proliferative tissue ventrally. (**C**) CT image of same horse and sites as in MRI image (**A**). (**D**) CT image of same horse and sites as in MRI image (**B**).

T1-weighted, T2W, T1-FS, PD images were used to grade articular process joint-related variables including synovial effusion, synovial membrane thickening, and synovial proliferation on the sagittal, dorsal, and axial planes. Synovial effusion, synovial membrane thickening, and synovial proliferation were each individually graded 0–4 (Table 2). A total MRI synovial tissue grade (0–12) was calculated for each articular process joint by adding the individual synovial effusion, synovial membrane thickening, and synovial proliferation grades. Grade 1–4 synovial effusion, synovial membrane thickening, and synovial proliferation are demonstrated in Figure 1, Figure 2 and Figure 3.

T1-weighted, T2W, T1-FS, PD images were also used to evaluate cervical vertebral nerve-related variables including cervical vertebral nerve identification, abnormality of cervical vertebral nerve size and shape including an increase or decrease in size and/or a misshapen nerve, and abnormality of cervical vertebral perineural tissue including alterations in the fat distribution and/or replacement with intermediate density and/or scar tissue(s) on sagittal, dorsal and axial planes. Cervical vertebral nerve identification, normality of cervical vertebral nerve size, and normality of cervical vertebral perineural tissues were all assessed as “yes” or “no”.

Confidence grading (Table 3) of synovial tissues and vertebral nerves was used to grade the observers’ confidence in their ability to identify these tissues distinctly on MRI. Synovial tissues and vertebral nerve identification were graded 0–3.

For all MRI evaluations, variables were not graded if image quality or artifacts interfered with assessment.

### 2.6. Equine Cervical CT Myelography Assessments

Corresponding regions to those graded on MRI were graded on CT myelography images, and only CT myelography images acquired in neutral position were used. Images were used to grade articular process joint-related variables on the sagittal, dorsal, and axial planes. Synovial tissues were graded 0–4 (Table 4). Although synovial effusion, membrane thickening, and proliferation were graded separately on MRI, synovial tissues were graded as a single entity because these tissues cannot be distinguished separately on CT. Hounsfield units of the parasagittal (lateral), axial (medial), and dorsal (dorsomedial) joint recesses [19] were also measured and recorded individually by each radiologist on the parasagittal, dorsal and axial planes.

Computed tomography images were also used to grade cervical vertebral nerve-related variables including cervical vertebral nerve identification, abnormality of cervical vertebral nerve size and shape, and abnormality of cervical vertebral perineural tissue on the sagittal, dorsal, and axial planes. Cervical vertebral nerve identification, normality of cervical vertebral nerve size, and normality of cervical vertebral perineural tissues were all assessed as “yes” or “no”. Hounsfield units of the presumptive cervical vertebral nerve tissue in the intervertebral foramen on the axial plane were measured individually by each radiologist.

Confidence grading (Table 3) of synovial tissue and vertebral nerves was used to grade the observers’ confidence in their ability to confidently identify these tissues distinctly on CT. Synovial tissues and vertebral nerve identification were graded 0–3.

Hounsfield units (HU) recorded were categorized for agreement calculations as 0—HU ≤ 0, 1—HU = 1–19, 2—HU = 20–300, 3—HU > 300.

For all CT evaluations, variables were not graded if image quality or artifacts interfered with assessment.

### 2.7. Statistical Analyses

Continuous data were expressed as mean ± standard deviation. The comparison of two measurement techniques, MRI versus CT, for the assessment of cervical articular process joint soft tissue normality, cervical vertebral nerve normality, and cervical spinal perineural tissue normality was performed using the McNemar test to test the difference between paired proportions. Comparisons of confidence grades of cervical articular process joint soft tissues and cervical vertebral nerves were performed using Bland–Altman plot differences. Comparison of Radiologist 1 versus Radiologist 2 for the assessment of Hounsfield unit measurements of soft tissues within cervical joint recesses and intervertebral foramen on CT was performed using an inter-rater agreement statistic (Kappa) to evaluate the agreement between two classifications. Comparisons of CT joint grade between cervical sites, MRI joint effusion between cervical sites, MRI synovial membrane thickening between cervical sites, MRI synovial proliferation between cervical sites, and MRI joint total grade between cervical sites were performed using the Wilcoxon rank sum test.

All the statistical analyses were performed using MedCalc^®^ software for Windows, version 19.4 (MedCalc Software Ltd., Ostend, Belgium). A *p*-value of <0.05 was considered significant.

## 3. Results

Cadaveric specimens from 18 athletic horses were included in the study including 16 warmbloods and two thoroughbreds. Horses included 15 geldings, two stallions and one mare ranging from 1 to 12 (mean 6.8) years of age.

Cervical (C) vertebral sites that were available and evaluated for both CT and MRI evaluations included five left and right C2-C3, for a total of 10 C2-C3 sites; seven left and right C3-C4, for a total of 14 C3-C4 sites; five left and right C4-C5, for a total of 10 C4-C5 sites; 12 left and right C5-C6, for a total of 24 C5-C6 sites; 18 left and right C6-C7, for a total of 36 C6-C7 sites; 16 left and right C7-T1, for a total of 32 C7-T1 sites.

Magnetic resonance imaging sequences were selected to optimize the conspicuity of the articular process joints and vertebral nerves based on their expected signal characteristics. Abnormalities within the articular process joints, particularly effusion and synovial membrane margins, were most clearly delineated on T2W and PD sequences. On these sequences, joint effusion was hyperintense and sharply contrasted with the hypointense synovial membrane and adjacent intermediate signal intensity adipose tissue, clearly defining the joint recess margins. Conversely, the T1W and PD images provided superior visualization of the vertebral nerves, which appeared with an intermediate signal intensity against the surrounding hyperintense adipose tissue. It is important to note that the overall conspicuity of both the vertebral nerves and articular process joint margins was dependent on the surrounding tissue and the presence of any pathology. In cases with abnormalities, the optimal sequence for structural identification was dictated by the specific nature of the abnormalities and associated changes in the characteristic signal pattern.

**Figure 4:**
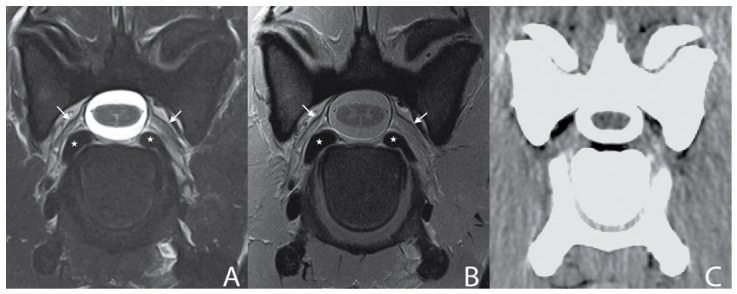
Comparison of MRI cervical vertebral nerve and perineural tissues and corresponding CT image. (**A**) T2W MRI with normal (left arrow) and abnormal (right arrow) cervical vertebral nerve and perineural tissue. (**B**) PD MRI with normal (left arrow) and abnormal (right arrow) cervical vertebral nerve and perineural tissue. In (**A**,**B**), postmortem air is seen in vasculature (stars) and abnormalities (right arrows) include thickening of the vertebral nerve and perineural soft tissue and loss of perineural fat. (**C**) CT image of same horse and site as in MRI images (**A**,**B**).

MRI was significantly superior to CT at evaluating cervical articular process joint soft tissues at C5-C6, C6-C7 and C7-T1 (Table 5). Figure 1, Figure 2 and Figure 3 demonstrate the varying grades of synovial effusion (Figure 1), membrane thickening (Figure 2), and proliferation (Figure 3) seen on axial T2W MRI images with corresponding comparative axial CT images. MRI was also significantly superior to CT at evaluating cervical vertebral nerves at C6-C7 (CVN 7) (Table 5) and cervical spinal perineural tissues at C5-C6 (Table 5). Figure 4 demonstrates normal and abnormal cervical vertebral nerves and perineural tissues on axial T2W and PD MRI images with corresponding comparative axial CT images. Both radiologists had significantly greater confidence in MRI than CT for identifying cervical articular process joint soft tissues and cervical vertebral nerves at all sites examined (Table 6).

Agreement between radiologists’ measurements of Hounsfield units on CT images was variable for all sites examined (Table 7). For measurements of the cervical vertebral nerve tissues in the intervertebral foramina on the axial plane, no to fair (−0.11–0.27) agreement was found. For measurements of the axial joint recess synovial tissues, no to good (−0.07–0.63) agreement in the parasagittal plane, poor to good (0.12–0.62) agreement in the axial plane, and poor to fair (0.18–0.36) agreement in the dorsal plane were found. Overall, synovial tissue in the axial plane had the best agreements between radiologists, and spinal nervous tissue had the worst agreements between radiologists.

MRI was able to identify significant differences between more sites in total synovial grades, synovial effusion, synovial membrane thickening, and synovial proliferation than CT identified between CT grades (Table 8). Generally, MRI grades were higher for more caudal than cranial cervical sites with the exception of C3-C4. For MRI synovial total grades, the lowest grade was found at C2-C3, and the highest grade was found at C6-C7. For MRI joint effusion grades, the lowest grade was found at C2-C3, and the highest grade was found at C6-C7. For MRI synovial membrane thickening grades, the lowest grade was found at C2-C3, and the highest grade was found at C3-C4. For synovial proliferation grades, the lowest grade was found at C2-C3, and the highest grade was found at C6-C7. MRI repeatedly identified significantly greater abnormalities at C3-C4, C6-C7, and C7-T1. CT identified a significantly greater synovial grade at C6-C7 compared to C4-C5 only.

## 4. Discussion

Magnetic resonance imaging was superior to CT myelography, and radiologists demonstrated clear confidence in MRI compared to CT myelography for identifying equine cervical vertebral nerve and synovial soft tissues. These findings were not surprising, as MRI has been well-documented in people and other species, including horses, to be the modality of choice for examining soft tissues. Contrast resolution of CT is poor and inadequate for thorough examination and identification of pathological changes associated with peripheral nerves and provides only indirect information regarding peripheral nerve disease [18,20]. In people, MRI is preferred specifically for imaging nervous tissues [21,22,23]. Prior to MRI, the evaluation of peripheral neuropathies relied on clinical examination and electrodiagnostic testing [20,24]. Multiple studies have shown that MRI neurography depicts nerve anatomy and pathology well and that findings substantially influence the management of patients with neuropathies [20,22,23,24]. In horses, it has been shown that cervical soft tissues such as the spinal cord, cerebrospinal fluid, adipose tissue in the epidural space, and intervertebral discs were more clearly identified with MRI, compared to CT [25].

Computed tomography cannot be used to distinguish nerves from surrounding isodense soft tissues. However, CT has been suggested as a useful screening tool for peripheral nerve injuries in people, as it can help identify potential contributing factors, such as bony abnormalities in close proximity to the nerve [21]. In horses, the cervical vertebral nerves are located within the intravertebral foramina, surrounded by fat and other soft tissues. The perineural fat is hypodense or hypoattenuating on CT and, when present, should theoretically be distinguishable from the isodense or isoattenuating cervical vertebral nerve and other perineural tissues, depending on the quality and spatial resolution of the study. Despite this potential for differentiation, CT myelography cases in this study revealed various abnormalities in the area of the cervical vertebral nerve and perineural tissues, including articular process enlargement, intervertebral foraminal narrowing, and articular process joint synovitis, suggesting possible nerve involvement. However, repeatable and definitive identification and differentiation of the cervical vertebral nerve tissue or detection of pathology were not possible on CT. In some sites, compression of fat and other tissues within the intravertebral foramina was observed as a reduction or loss of the hypoattenuating fat. Where significant synovitis was present, a general amorphous isoattenuating soft tissue material was identified extending from the axial joint recesses into the foramina. Unlike MRI, CT myelography could not be used to definitively and consistently distinguish between the different tissues, including fat, cervical vertebral nerve, synovial membrane, and synovial effusion.

Hounsfield units provide a quantitative scale to characterize tissues on CT according to their radiodensity relative to water and have been shown to aid in distinguishing fat versus soft tissue, fluid versus blood, and benign versus malignant lesions, and identifying calcification and acute hemorrhage [26,27]. Although they may vary between CT units based on the individual scanner, protocol used, and patient factors, typical HU ranges exist for particular tissues. Unlike fat which is hypodense and has a corresponding range of negative HU values, peripheral nervous tissue, similar to other soft tissues such as muscle and connect tissues, is isodense and therefore cannot be distinguished from adjacent soft tissues [26,27]. Agreement between radiologists’ HU measurement ranges was variable and poorest for measurements of tissues within the IVF where the cervical vertebral nerve should have been located. This finding further supports their lack of confidence in identifying the cervical synovial structures, particularly the cervical vertebral nerves. While CT imaging of peripheral nerves is flawed due to the lack of contrast resolution, there are techniques that have been used in human medicine that improve CT identification of peripheral nerves. The most successful comprises injection of a tri-iodinated lidocaine contrast agent to anesthetize and enhance visibility of peripheral nerves, primarily for intraoperative uses, and has been shown to be useful for preoperative planning and intraoperative nerve protection to decrease surgical nerve injuries in people [28]. Although local anesthesia of cervical vertebral nerves in horses could risk local blockade to the nerve and subsequently sensory and motor input to the limb(s), which would be undesirable for recovery from general anesthesia, a similar contrast-labeled agent might aid in better imaging these nerves on CT in horses.

All sites evaluated with MRI had evidence of synovial-related abnormalities. Significantly greater synovial-related abnormalities were identified at articular process joints (APJs) of C3-C4, C6-C7, and C7-T1 compared to other sites, with the greatest total grades at C7-T1, C6-C7, and C3-C4 respectively. These findings are similar to previous studies that have reported high incidences of osteoarthrosis (OA) in horses undergoing cervical CT, with 50/51 horses having one or more APJs affected in one study of horses with vertebral lesions, and 83% in another study of horses undergoing CT for clinical signs of cervical disease [16,17,29]. These studies also reported more severe and frequent OA findings in the caudal APJs, with C6-C7, C5-C6, C4-C5, and C3-C4 being common sites [16,17,29].

The majority of horses in this study were warmblood and thoroughbred males, with most being geldings. While this is similar to the hospital caseload demographics for English sport horses, the percentage of males and warmblood horses is greater than the hospital general population. This may be due to an overrepresentation of warmbloods, geldings and stallions having cervical disease in comparison to others. Levine et al. found geldings were 2.0 times and stallions were 2.4 times more likely to be diagnosed with cervical vertebral compressive myelopathy compared to mares, with thoroughbreds, Tennessee Walking Horses, and warmbloods being overrepresented [30]. Similar demographic trends have also been reported in horses evaluated for cervical pain and stiffness [2].

Limitations of this study include the variability of cervical disease in the horses included and smaller sample sizes of cervical sites that were not considered clinically related to the horses’ cervical disease. Magnetic resonance imaging of all cervical sites was not performed. Sites that were found to have clinically relevant abnormalities on CT myelography were imaged by MRI, and additional sites were imaged when time permitted, but this was uncommon. Had all sites been imaged on both CT myelography and MRI, the sufficient sample size should have allowed comparisons that achieved statistical significance and supported MRI as superior to CT myelography at all sites. Additionally, an inherent limitation of CT is the inability to distinguish and grade synovial effusion, membrane thickening, and proliferation separately and then compare individually to MRI. Although this is a limitation, it is also a purpose for performing this study to validate and increase awareness of this limitation.

## 5. Conclusions

Magnetic resonance imaging should be considered the gold standard for imaging cervical vertebral nerves and synovial soft tissues in horses, as it is for other species. Unfortunately, currently, antemortem evaluation of the mid and caudal cervical spine of most adult horses with MRI is not possible due to unit configurations and bore sizes. With increased consideration for equine cervical disease, demand and desire for this imaging is growing, although it is unlikely in the near future. Computed tomography with or without myelography remains the best option for imaging the cervical spine of horses with cervical disease. When imaging the cervical spine of horses with CT myelography, caution should be exercised when interpreting findings of cervical synovial soft tissues and cervical vertebral nerves.

## Figures and Tables

**Table 1:** Magnetic resonance imaging parameters.

Pulse Sequence	Field of View (cm)	Echo Train	NEX	TR (ms)	TE (ms)	Slice Thickness (cm)	Spacing (mm)	In-Plane Resolution (mm)	Matrix
Sagittal T1	48 × 48	3	2	550–700	8.7–18.7	3–5	1	1.875 × 1.875	256 × 256
Sagittal T2	48 × 48	15	2	2766.7–6273	83.1–128.3	4–5	1	1.875 × 1.875	256 × 256
Axial T1	30 × 29.2		2	529–930	10–20	4–5	2.5	1.17 × 1.17	256 × 256
Axial T2	30 × 29.2	15	2	4000–11,283.3	103.4–120	4–5	2.5	1.17 × 1.17	256 × 256
Dorsal T1	48 × 48	3	2	500–966.7	10–20	4–5	1	1.875 × 1.875	256 × 256
Dorsal T1 FS	40 × 40	4	2	715	10	5	0.5	1.875 × 1.875	256 × 224
Axial PD	18 × 18	4	1	5264	18	5	0.5	1.875 × 1.875	336 × 288

**Table 2:** Magnetic resonance imaging synovial variable grading schemes. Synovial effusion, membrane thickening, and proliferation were each graded (0–4) individually.

Grade	Severity	Effusion	Membrane Thickening	Proliferation
0	Normal	No effusion present	No membrane thickening	No proliferation
1	Mild	Effusion identified between cranial and caudal articular processes, does not extend into cranial joint recess or intervertebral foramen	Mild membrane thickening	Mild proliferation
2	Mild to Moderate	Effusion extends ventrally, causing axial expansion of synovial membrane	Mild to moderate membrane thickening	Mild to moderate proliferation
3	Moderate	Effusion extends into cranial joint recess and/or shows increased axial expansion of synovial membrane	Moderate membrane thickening	Moderate proliferation
4	Marked	Marked effusion in cranial joint recess and/or extending adjacent to spinous process and/or into intervertebral foramen	Marked membrane thickening	Marked proliferation

**Table 3:** Confidence grading scheme for synovial tissues and vertebral nerves.

Grade	Confidence Level	Description
0	Not Confident	Observer is unable to identify the tissue distinctly
1	Minimally Confident	Observer has limited ability to identify the tissue; identification is unclear
2	Moderately Confident	Observer can identify the tissue with some certainty, but not definitively
3	Confident	Observer is clearly able to identify the tissue with high certainty

**Table 4:** Computed tomography synovial tissue grading scheme.

Grade	Severity	Description
0	Normal	No synovial effusion/tissue thickening
1	Mild	Mild synovial effusion/tissue thickening
2	Mild to Moderate	Mild to moderate synovial effusion/tissue thickening
3	Moderate	Moderate synovial effusion/tissue thickening
4	Marked	Marked synovial effusion/tissue thickening

**Table 5:** Comparison of magnetic resonance imaging versus computed tomography for assessment of normality. * Denotes statistical significance.

Sites	Difference	95% CI	*p*
**Cervical Articular Process Joint Soft Tissues**			
C2-C3	50%	19.01–80.99	0.06
C3-C4	21%	−0.066–42.92	0.25
C5-C6	25%	7.68–42.32	0.03 *
C6-C7	19%	6.52–32.37	0.02 *
C7-T1	53%	35.83–70.42	<0.001 *
**Cervical Vertebral Nerves**			
C3-C4 (CVN 4)	−43%	−79.52–6.20	0.25
C4-C5 (CVN 5)	−14%	−40.21–11.64	1.00
C5-C6 (CVN 6)	−5%	−22.97–12.45	1.00
C6-C7 (CVN 7)	−36%	−54.82–17.18	0.003 *
C7-T1 (CVN 8)	−26%	−46.12–6.52	0.06
**Cervical Spinal Perineural Tissues**			
C3-C4	9%	−30.39–48.57	1.00
C4-C5	30%	−9.69–69.69	0.38
C5-C6	36%	16.26–56.47	0.008 *
C6-C7	0%	−14.00–14.00	1.00
C7-T1	0%	−24.00–24.00	1.00

**Table 6:** Comparisons of confidence scores. * Denotes statistical significance.

Site	Difference	95% CI	*p*
**Cervical Articular Process Joint Soft Tissues**			
C2-C3	−83%	−95.90–70.77	<0.001 *
C3-C4	−74%	−82.00–65.61	<0.001 *
C4-C5	−67%	−66.67–66.67	<0.001 *
C5-C6	−88%	−96.38–78.62	<0.001 *
C6-C7	−69%	−75.25–62.12	<0.001 *
C7-T1	−83%	−90.16–76.51	<0.001 *
**Cervical Vertebral Nerves**			
C2-C3 (CVN 3)	−83%	−98.23–68.44	<0.001 *
C3-C4 (CVN 4)	−81%	−133.35–95.01	<0.001 *
C4-C5 (CVN 5)	−77%	−88.19–65.15	<0.001 *
C5-C6 (CVN 6)	−67%	−75.95–57.39	<0.001 *
C6-C7 (CVN 7)	−70%	−78.22–62.73	<0.001 *
C7-T1 (CVN 8)	−78%	−85.35–70.90	<0.001 *

**Table 7:** Comparison of Radiologist 1 versus Radiologist 2 for assessment of Hounsfield unit measurements of soft tissues within cervical joint recesses and intervertebral foramina on computed tomography.

Site	Weighted Kappa	Strength of Agreement	95% CI	Standard Error
**Parasagittal Joint Recesses (Synovial Tissue and Fluid)**				
C2-C3	0.63	Good	0.31–0.96	0.16
C3-C4	0.49	Moderate	0.16–0.81	0.16
C4-C5	−0.07	No agreement	−0.43–0.29	0.18
C5-C6	0.42	Moderate	0.17–0.68	0.13
C6-C7	0.25	Fair	−0.02–0.52	0.14
C7-T1	0.40	Fair	0.16–0.65	0.12
**Axial Joint Recesses (Synovial Tissue and Fluid)**				
C2-C3	0.52	Moderate	0.12–0.93	0.20
C3-C4	0.23	Fair	−0.17–0.64	0.21
C4-C5	0.12	Poor	−0.29–0.54	0.21
C5-C6	0.62	Good	0.38–0.87	0.13
C6-C7	0.18	Poor	−0.07–0.42	0.13
C7-T1	0.39	Fair	0.13–0.66	0.13
**Dorsal Joint Recesses (Synovial Tissue and Fluid)**				
C2-C3	0.19	Poor	−0.23–0.60	0.21
C3-C4	0.35	Fair	−0.02–0.72	0.19
C4-C5	0.24	Fair	−0.32–0.80	0.29
C5-C6	0.18	Poor	−0.09–0.46	0.14
C6-C7	0.24	Fair	−0.04–0.51	0.14
C7-T1	0.36	Fair	0.12–0.60	0.12
**Intervertebral Foramina (Vertebral Nerve Tissue)**				
C4-C5 (CVN 5)	0	No agreement	0.00 to 0.00	0
C5-C6 (CVN 6)	−0.11	No agreement	−0.22 to 0.01	0.06
C6-C7 (CVN 7)	0.05	Poor	−0.24 to 0.34	0.15
C7-T1 (CVN 8)	0.27	Fair	0.01 to 0.53	0.13

**Table 8:** Comparisons of grades between cervical sites. * Denotes the higher median value. ** Denotes statistical significance.

Site	Lower Median	Higher * Median	95% CI	*p*
**MRI Joint Effusion**				
C2-C3 vs. C3-C4 *	1	2	0.00–2.00	0.02 **
C2-C3 vs. C5-C6 *	2	3	−1.00–3.00	0.13
C2-C3 vs. C6-C7 *	1	2	0.00–2.00	0.03 **
C2-C3 vs. C7-T1 *	1	2	0.00–1.00	0.05 **
C3-C4 * vs. C4-C5	2	3	−1.00–0.00	0.03 **
C3-C4 vs. C5-C6	3	3	−1.00–0.00	0.16
C3-C4 vs. C6-C7	2.5	2.5	−1.00–0.50	0.45
C3-C4 * vs. C7-T1	2	2.5	−1.50–−0.50	0.01 **
C4-C5 * vs. C5-C6	2	2.5	−1.50–0.50	0.32
C4-C5 vs. C6-C7 *	2	2.5	−0.50–0.50	0.65
C4-C5 * vs. C7-T1	1.5	2	−1.50–0.50	0.01 **
C5-C6 vs. C6-C7	3	3	−0.50–0.50	0.50
C5-C6 vs. C7-T1	2	2	−1.00–0.00	0.22
C6-C7 * vs. C7-T1	2	3	−1.00–−0.50	<0.001 **
**MRI Synovial Membrane Thickening**				
C2-C3 vs. C3-C4 *	1	2	0.50–2.00	0.01 **
C2-C3 vs. C6-C7 *	1	2	0.00–2.00	0.05 **
C2-C3 vs. C7-T1 *	1	1.5	0.00–1.00	0.25
C3-C4 * vs. C4-C5	2	3	−1.50–0.50	0.16
C3-C4 * vs. C5-C6	2	3	−1.00–0.00	0.05 **
C3-C4 vs. C6-C7	2	2	−1.00–0.50	0.24
C3-C4 * vs. C7-T1	1.5	2	−1.50–−0.50	0.01 **
C4-C5 * vs. C5-C6	2	2.5	−1.00–0.50	0.65
C4-C5 vs. C6-C7	2	2	−1.50–1.00	0.31
C4-C5 * vs. C7-T1	1.5	2	−1.50–0.50	0.19
C5-C6 vs. C6-C7	2	2	0.00–0.50	0.41
C5-C6 vs. C7-T1	2	2	−0.50–0.50	1.00
C6-C7 vs. C7-T1	2	2	−1.00–0.00	0.10
**MRI Synovial Proliferation**				
C2-C3 vs. C5-C6 *	1	2	−1.00–1.00	0.65
C2-C3 vs. C6-C7 *	1	2	0.50–1.00	0.01 **
C3-C4 vs. C4-C5	1	1	−1.00–1.00	0.71
C3-C4 vs. C5-C6 *	1	2	−1.00–0.50	0.59
C3-C4 vs. C6-C7 *	1	2	0.00–1.00	0.19
C3-C4 vs. C7-T1	1	1	−1.00–0.50	0.50
C4-C5 vs. C5-C6 *	1	1.5	−0.50–1.00	0.65
C4-C5 vs. C6-C7 *	1	2	0.00–1.50	0.08
C4-C5 vs. C7-T1	1	1	−1.00–1.00	0.56
C5-C6 vs. C6-C7	2	2	0.00–1.00	0.09
C5-C6 vs. C7-T1	2	2	0.00–1.00	0.18
C6-C7 vs. C7-T1	2	2	−0.50–0.50	0.74
**MRI Synovial Total Grades**				
C2-C3 vs. C3-C4 *	2.5	5.5	1.50–7.00	0.01 **
C2-C3 vs. C6-C7 *	3.5	7.7	1.50–5.50	0.01 **
C2-C3 vs. C7-T1 *	3.5	4.5	0.00–2.50	0.09
C3-C4 * vs. C4-C5	4	7	−3.00–0.50	0.11
C3-C4 * vs. C5-C6	6.5	7	−2.50–0.00	0.08
C3-C4 vs. C6-C7 *	6.5	7	−1.50–1.00	0.78
C3-C4 * vs. C7-T1	4	6.5	−3.50–-0.50	0.01 **
C4-C5 * vs. C5-C6	6	7	−2.00–1.00	0.55
C4-C5 vs. C6-C7 *	5.5	7	−1.50–1.50	0.89
C4-C5 * vs. C7-T1	4	5.5	−3.50–0.50	0.09
C5-C6 vs. C6-C7 *	6	7	−0.50–1.50	0.24
C5-C6 vs. C7-T1 *	6	7	−1.50–1.00	0.64
C6-C7 vs. C7-T1	7	7	−2.00–−0.50	<0.001 **
**CT Synovial Grades**				
C3-C4 vs. C6-C7	2	2	0.00–1.00	0.16
C4-C5 vs. C6-C7 *	2	3	0.50–2.00	0.01 **
C4-C5 vs. C7-T1	2	2	−1.00–1.00	1.00
C5-C6 vs. C6-C7	2	2	0.00–1.00	0.08
C5-C6 vs. C7-T1	2	2	−1.50–0.50	0.32
C6-C7 vs. C7-T1	2	2	−1.00–1.00	0.60

## Data Availability

The data presented in this study are available on request from the corresponding author due to privacy regulations.

## Abbreviations

The following abbreviations are used in this manuscript:

C	Cervical
CT	Computed tomography
CVN	Cervical vertebral nerve
MRI	Magnetic resonance imaging
HU	Hounsfield unit
CSF	Cerebrospinal fluid
T1W	T1-weighted
T2W	T2-weighted
T1-FS	T1-weighted with fat saturation
PD	Proton density
DICOM	Digital Imaging and Communications in Medicine
IVF	Intervertebral foramen/foramina
